# A mixed methods study of Aboriginal health workers’ and exercise physiologists’ experiences of co-designing chronic lung disease ‘yarning’ education resources

**DOI:** 10.1186/s12889-023-15508-y

**Published:** 2023-03-31

**Authors:** David P. Meharg, Sarah M. Dennis, Justin McNab, Kylie G. Gwynne, Christine R. Jenkins, Graeme P. Maguire, Stephen Jan, Tim Shaw, Zoe McKeough, Boe Rambaldini, Vanessa Lee, Debbie McCowen, Jamie Newman, Scott Monaghan, Hayley Longbottom, Sandra J. Eades, Jennifer A. Alison

**Affiliations:** 1 Faculty of Medicine and Health, Sydney School of Health Sciences, The University of Sydney, Western Avenue, Camperdown, NSW 2006 Australia; 2 Poche Centre for Indigenous Health, The University of Sydney, Camperdown, NSW 2006 Australia; 3 South Western Sydney Local Health District, Liverpool, NSW 2170 Australia; 4 Ingham Institute for Applied Medical Research, Liverpool, NSW 2170 Australia; 5 Reproduction and Perinatal Centre, Faculty of Medicine and Health, The University of Sydney, Sydney, NSW 2006 Australia; 6 Charles Perkins Centre, The University of Sydney, Sydney, NSW 2006 Australia; 7 Faculty of Medicine, Health and Human Sciences, Macquarie University, North Ryde, NSW 2109 Australia; 8 The George Institute for Global Health, Newtown, NSW 2042 Australia; 9 University of New South Wales, Sydney, Kensington, NSW 2052 Australia; 10 Curtin Medical School, Faculty of Health Sciences, Curtin University, Bentley, WA 6102 Australia; 11 Faculty of Medicine and Health, School of Medical Sciences, The University of Sydney, Camperdown, NSW 2006 Australia; 12 Armajun Aboriginal Health Service, Inverell, NSW 2360 Australia; 13 Orange Aboriginal Medical Service, Orange, NSW 2800 Australia; 14 Bulgarr Ngaru Medical Aboriginal Corporation, Grafton, NSW 2460 Australia; 15 Waminda South Coast Women’s Health and Welfare Aboriginal Corporation, Nowra, NSW 2541 Australia; 16 Centre for Epidemiology and Biostatistics, The School of Population and Global Health, The University of Melbourne, Carlton South, Victoria, Australia; 17 Sydney Local Health District, Camperdown, NSW 2050 Australia

**Keywords:** Aboriginal health worker, Chronic obstructive pulmonary disease, Aboriginal pedagogy, Education and pulmonary rehabilitation

## Abstract

**Background:**

Despite the high incidence of chronic obstructive pulmonary disease (COPD) in Aboriginal communities in Australia, Aboriginal Health Workers (AHWs) have limited knowledge about effective management.

**Aim:**

To evaluate an online education program, co-designed with AHWs and exercise physiologists (EPs) or physiotherapists (PTs), to increase knowledge about COPD and its management.

**Methods:**

AHWs and EPs from four Aboriginal Community Controlled Health Services (ACCHS) were recruited. An Aboriginal researcher and a physiotherapist experienced in COPD management and pulmonary rehabilitation (PR) delivered seven online education sessions. These sessions used co-design principles and an Aboriginal pedagogy framework ‘8 Ways of learning’, which incorporates Aboriginal protocols and perspectives to realign teaching techniques and strengthen learning outcomes. Topics covered were: How the lungs work; What is COPD; Medications and how to use inhalers and COPD Action Plans; Why exercise is important; Managing breathlessness; Healthy eating; Managing anxiety and depression. After each session, AHWs with support from EPs, co-designed education ‘yarning’ resources using Aboriginal ways of learning to ensure topics were culturally safe for the local Aboriginal community and practiced delivering this at the following session. At the end of the program participants completed an anonymous online survey (5-point Likert scale) to assess satisfaction, and a semi-structured interview about their experience of the online education.

**Results:**

Of the 12 participants, 11 completed the survey (7 AHWs, 4 EPs). Most (90%) participants strongly agreed or agreed that the online sessions increased knowledge and skills they needed to support Aboriginal patients with COPD. All (100%) participants felt: their cultural perspectives and opinions were valued and that they were encouraged to include cultural knowledge. Most (91%) reported that delivering their own co-designed yarning scripts during the online sessions improved their understanding of the topics. Eleven participants completed semi-structured interviews about participating in online education to co-design Aboriginal ‘yarning’ resources. Themes identified were: revealing the Aboriginal lung health landscape; participating in online learning; structuring the online education sessions; co-designing with the facilitators.

**Conclusions:**

Online education using co-design and 8 Ways of learning was rated highly by AHWs and EPs for improving COPD knowledge and valuing cultural perspectives. The use of co-design principles supported the cultural adaptation of COPD resources for Aboriginal people with COPD.

**Trial registration:**

PROSPERO (registration number: CRD42019111405).

**Supplementary Information:**

The online version contains supplementary material available at 10.1186/s12889-023-15508-y.

## Introduction

Aboriginal and Torres Strait Islander people, the Indigenous peoples of Australia (hereafter respectfully referred to as Aboriginal people), have a higher burden of chronic obstructive pulmonary disease (COPD) than other Australians [1–4], which could be addressed by improved access to primary care services [5]. Pulmonary rehabilitation (PR) is a key component of effective COPD management [6]. PR is an evidence-based program, typically conducted for one hour, twice a week for 8 weeks. PR programs consist of individually tailored exercise training prescribed by a physiotherapist (PT) or exercise physiologist (EP), and education sessions related to COPD management [7]. Most PR programs in Australia are provided by state-funded local health services in hospital outpatient settings [8]. Access to and uptake of these mainstream PR programs by Aboriginal people is low [9], largely due to mainstream health services being perceived as culturally unsafe [10].

Aboriginal Community Controlled Health Services (ACCHS) are incorporated organisations, governed by a Board consisting of local Aboriginal community members. ACCHS were established to address the inadequacies of mainstream health services to deliver culturally safe care for Aboriginal people [11, 12]. ACCHS operate in dynamic environments and deliver holistic and effective primary health care in innovative ways that respond to local health priorities [13]. Although ACCHS deliver a range of chronic care services, PR is not typically offered as core business and there is no specific funding mechanism for PR in these settings [14]. There has been little published about the outcomes of PR for Aboriginal people with COPD, with only one study of PR provided by an ACCHS reporting that Aboriginal participants with chronic respiratory or heart disease significantly improved functional exercise capacity and quality of life [9]. The reportedly low level of knowledge and confidence of the Australian rural and remote Aboriginal and non-Aboriginal health workforce around the provision of PR [15] may impact delivery of this service.

Enhancing a capable and trained Aboriginal health workforce has shown to improve health care access and outcomes for Aboriginal people and positively contribute to the performance, quality and deliverable outcomes of the Aboriginal primary health sector [5]. ACCHS function at the cultural interface between Western medical delivery and Aboriginal ways of knowing, being and doing. Therefore, building an Aboriginal health workforce that has both professional and cultural competence is a priority [5, 16, 17]. In Australia, Canada, New Zealand and the United States, Indigenous health care workers perform critical functions as cultural brokers in program planning, service delivery and care by applying clinical and socio-cultural skills to improve patients’ access to culturally safe care [17, 18]. An Australian narrative review identified strategies to develop and maintain a skilled rural and remote health workforce and revealed the importance of Aboriginal and non-Aboriginal staff working together, with Aboriginal staff being supported to use their specialised skill sets and knowledge of the local Aboriginal community needs to effectively implement health programs [19]. The importance of designing and delivering training focused on the specific needs of both the Aboriginal workforce and the Aboriginal community receiving the program or care was also highlighted [19].

The ‘8 Ways of learning’ is an Aboriginal pedagogy framework and was an initiative of the NSW Department of Education that engaged Aboriginal knowledge holders in its development. 8 Ways of learning incorporates cultural protocols and Aboriginal perspectives to realign teaching techniques and approaches, and to strengthen engagement and outcomes for Aboriginal learners [20]. Fundamental to this framework is prioritising people and relationships and integrating Aboriginal ways of knowing, being and doing [20–22]. The cultural protocols include the following 8 ways: *Deconstruct/reconstruct* (dismantling and reassembling approaches using local Aboriginal world views); *Community Links* (identification of key community stakeholders to be consulted about projects); *Learning maps* (exploring and visualising concepts and strategies); *Story Sharing* (clarifying and sharing content verbally); *Symbols and images* (explaining concepts using artwork and visual methods); *Land links* (relating learning to spaces, places and the living landscape); *Non-verbal* (using hands-on activities and body language, including silence); and *Non-linear* (non-consequential and indirect orientation of learning) [20, 21].

While face-to-face experiential learning and multi-faceted workforce strategies have been shown to be effective in building knowledge, confidence and skills of the Aboriginal health workforce [17], the impact of the COVID-19 pandemic and travel-related restrictions meant sectors such as education and health needed to pivot to virtual care and online learning [23–27]. The pandemic provided an opportunity to reflect on the design of online training, the learning needs of the Aboriginal health workforce and scaffolding strategies to build knowledge and autonomy as well as teaching approaches [28], such as the incorporation of 8 Ways of learning and yarning. Yarning for Aboriginal people is a cultural conversational process of sharing stories and experiences, thoughts and ideas in culturally safe environments which centres around Aboriginal ways of knowing, being and doing [29–31].

The aim of this study was to explore the experiences of AHWs, EPs and PTs within ACCHS who attended online education sessions using co-design principles and 8 Ways of learning to enhance workforce capacity and provide culturally safe education as part of a PR program for Aboriginal people with COPD. The study used participatory action research which encourages self-reflective enquiry and shares power and decision making with participants in the design and/or delivery of programs [23] using co-design principles [24], which aligns well with 8 Ways of learning. Aboriginal health projects using participatory action research, guided by co-design principles, and incorporating Aboriginal perspectives have effectively engaged stakeholders and produced measurable outcomes [25].

## Methods

### Context and settings

This mixed methods study was a component of the overarching Breathe Easy Walk Easy Lungs for Life (BE WELL) project, evaluating the implementation of PR within ACCHS. The study is approved by the Aboriginal Health & Medical Research Council of NSW Human Resource Ethics Committee (HREC 1261/17).

For the overall BE WELL project, the research team contacted all NSW-based ACCHS by letter inviting them to join the study. Four ACCHS classified as Rural Zone Codes 3–5, agreed to participate [32]. The BE WELL project protocol is published [33], and the trial is registered with the Australian New Zealand Clinical Trials Registry (ANZCTR): ACTRN12617001337369 [34].

### Participants

Study participants were staff from four ACCHS responsible for local BE WELL implementation. These were the EPs or PTs providing the exercise prescription and training and the AHWs providing education about COPD within the BE WELL PR programs. These staff (EPs/PTs and AHWs) were invited to attend a two-day face-to-face BE WELL Workshop which concentrated on patient assessment and the exercise training component of PR. Evaluation of the two-day workshop will be conducted separately. To provide training around the education component of PR, staff were invited to attend additional face-to-face training to build their COPD management knowledge and co-design the education (yarning) material. Due to COVID-19 travel-related restrictions in 2020, the additional face-to-face training was delivered online. Each of the four ACCHS negotiated the timing and frequency of the online education sessions around their existing responsibilities. All participants provided informed consent.

### Online education

Seven topics were included in the online education sessions. These were: How the lungs work; What is COPD; Medications and how to use inhalers and COPD Action Plans; Why exercise is important; Managing breathlessness; Healthy eating; Managing anxiety and depression. All sessions were delivered via Zoom using interactive internet-based video conferencing (also known as Voice over Internet Protocol (VoIP)-mediated technologies) with participant and facilitator discussion [35]. Sessions were between 30–60 min depending on participants’ preference. Sessions commenced with an Acknowledgment of Country and introductions. Each session was designed to incorporate 8 Ways of learning [20, 21] and co-design principles [36]. For each topic, the facilitator prepared and presented brief PowerPoint slides of the main topic points and, depending on the topic, sessions would include story sharing between participants stimulated by videos of patients with lung disease or demonstrations of the correct use of inhalers. Images, symbols, artwork, metaphors and analogies, as well as links to the living landscape, such as trees and river systems and meeting places were used to explain how the lungs work. Resources such as diagrams, booklets, medication charts, PowerPoint slide notes and session planning tools were provided. Examples of questions that the AHWs could ask patients to stimulate yarning around each of the topics were suggested and further developed. Following each session, AHWs with support from EPs/PTs, co-designed education ‘yarnings’ to deliver with their community. The yarnings used 8 Ways of learning and incorporated simple terms, phrases and words from the local Aboriginal languages to ensure each topic was engaging, relevant and culturally safe for each Aboriginal community. The yarning scripts developed by the AHW were used as a tool to clearly outline their yarning approach for each topic, key conversational points, stimulus questions and learning aides, such as pictorial or physical resources to be used.

### Outcome measures

After completing all the online education sessions, participants were invited via email to complete an anonymous online survey. The survey consisted of 19 questions, 16 of which used a 5-point Likert scale and three were free text. See Supplementary Survey, Additional file 1. Participants were also invited to participate in a semi-structured interview about their experience of the online education. See Supplementary Interview questions, Additional file 2.

Interviews were conducted using VoIP technologies by an investigator (KG) experienced in qualitative health research with Aboriginal participants, and who had not been involved in the design or delivery of the online education sessions. Interviews were conducted between November and December 2020. Recruitment ceased after all available AHW/EPs who participated in the online education sessions attended an interview. Sufficient data were collected to support saturation allowing for broad and deep insights related to the research question. A professional transcription service transcribed the audio recordings verbatim into text. Transcriptions were de-identified to ensure confidentiality of participants. DM and JA reviewed transcriptions against audio recordings for accuracy.

### Data analysis

Survey data were analysed using descriptive statistics within IBM SPSS statistics version 27. The questions with the 5-point Likert scale were given numerical values of -2 (strongly disagree/never/very poor), -1 (disagree/rarely/poor), 0 (unsure/sometimes/average), 1 agree/mostly/good), 2 (strongly agree/always/very good). Interview data were managed using NVivo (QSR International Pty Ltd 2020).

Reflexive thematic data analysis was applied within a framework of four central areas of AHW and EP experience participating in the online education sessions and co-designing ‘yarning’ resources, and their perception of patient experience of access to care and health literacy of COPD [37].

Thematic analysis used the six phases outlined by Braun and Clarke consisting of familiarisation, generating codes, searching for themes, reviewing potential themes, naming the themes and producing the report [38]. Familiarisation was facilitated by (DM, JA, SD) listening to the audio recordings and reading and rereading the transcriptions, taking notes and examining the data for meaning. Following familiarisation of the data, codes were defined inductively by (DM, JA, SD). Assumptions about code generation were checked with SD as the experienced qualitative researcher, with the perspectives of the Aboriginal researcher (DM) given deep consideration relating to their Aboriginal socio-cultural meaning. Final themes were generated based on central concepts and consensus reached by authors (DM, JA, SD, JMc).

Authors (DM and JA) remained conscious of reflexivity and the impact of their perspectives (professional, personal and cultural) during study implementation, data collection, analysis and presentation of the findings to strengthen rigour, trustworthiness and overall quality of the study. Quality of coding and analysis of the data was strengthened by authors (SD and JMc) through enquiry and verification of the inductive coding framework and coded text with authors (DM and JA) [37, 39].

## Results

Twelve participants, (eight AHWs and four EPs) from four NSW-based ACCHS implementing BE WELL attended online education sessions between July and December 2020 (Table 1). At the time of the online education, no PTs were employed by ACCHS. The online education sessions were delivered to each of the four ACCHS separately, with timing of sessions based on their existing clinical responsibilities. Session length was between 30–60 min and sessions were delivered over 5 to 11 weeks.

**Table 1:** Participant demographics

Characteristics		*N* = 12
*Completed survey and interview*		
Survey n (%)		11^a^ (92%)
Interview n (%)		11^b^ (92%)
*Ethnicity (Profession)*		
Aboriginal (AHW)		8
Non-Aboriginal (EP)		4
*Gender*		
Female:Male (n)		9:3
*Years working in current position (AHWs and EPs)*		
< 1 year		3
1–2 years		4
2–3 years		1
3–4 years		2
5 + years		2
*Years working in current position*		
Profession	Range	Mean *(SD)*
AHW	0.4—4.4	1.7 (*1.6*)
EP	1—10	5.2 (*4.5*)

*AHW* Aboriginal Health Worker, *EP* Exercise Physiologist

^a^ One AHW (male) did not complete the survey

^b^One AHW (female) did not complete the interview

### Survey

**Fig. 1:**
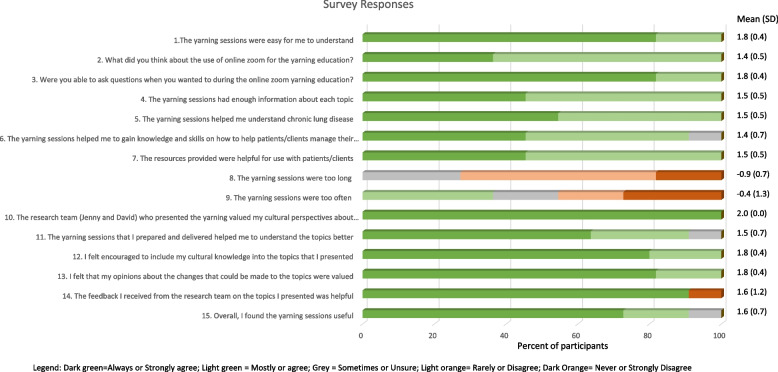
Survey responses

Eleven, (7 AHW and 4 EPs) completed the survey (Table 1). All (100%) participants Strongly Agreed or Agreed the online sessions were easy to understand (mean (SD)) (1.8 (0.4)), had enough information about each topic (1.5 (0.5)), helped them understand COPD (1.5 (0.5)), and that their cultural perspectives (2.0 (0.0)) and opinions (1.8 (0.4)) were valued and that the resources were helpful for patients (1.5 (0.5)) (Fig. 1).

Almost all (91%) participants Strongly Agreed or Agreed the yarning sessions enabled them to gain knowledge and skills on how to help patients manage COPD (1.4 (0.7)). Almost all (91%) participants Strongly Agreed that the feedback received on the topics they presented was helpful (1.6 (1.2)).

All (100%) participants rated using VoIP technologies for the yarning education as Very Good or Good (1.4 (0.5)). All (100%) participants were Always or Mostly able to ask questions during the online sessions (1.8 (0.4)). Almost all (91%) participants Always and Mostly felt that the yarning sessions they prepared and delivered helped them to understand the topics better (1.5 (0.7)), that they were encouraged to incorporate their cultural knowledge into the topics they presented (1.8 (0.4)), and overall, that the sessions were useful (1.6 (0.7)). The majority (73%) of participants Strongly Disagreed or Disagreed that the sessions were too long (-0.9 (0.7)). Almost half (45%), Strongly Disagreed or Disagreed that the sessions were too often (-0.4 (1.3)), and 36% Agreed (Fig. 1).

Participants ranked the seven education topics from most to least useful. The most useful topic was *How the lungs work* and the least useful was *Healthy Eating* (Table 2). Free-text feedback consistently reported that responding to this question was difficult as participants found each topic was equally important.

**Table 2:** Ranking of the usefulness of the seven online education topics

Most Useful Topics				Least Useful Topics		
*1*^*st*^ *How the lungs work*	*2*^*nd*^ *What is COPD*	*3*^*rd*^ *Managing breathlessness*	*4*^*th*^ *Medications and how to use inhalers and COPD* *Action Plans*	*5*^*th*^ *Managing Anxiety & Depression*	*6*^*th*^ *Why exercise is important*	*7*^*th*^ *Healthy Eating*

Participants also responded to three open ended questions: What participants liked most; What needed improving; Any additional comments. Participants reported they liked learning about the function of the lung and COPD the most. The sessions increased their knowledge about how to present information and support patients with COPD in a culturally safe way.

*“It really helped my team become more confident with delivering the content during the yarnings. Culturally I got a lot out of it.” (EP_02)*

Several participants reported that they valued the structure of the weekly topics and presenting their own yarnings.

“*The whole session was great; everything was presented well. Yarning sessions are great, very helpful. Learnt more with the weekly sessions.” (AHW_11)*

Suggested improvements included strengthening the formal structure of the first few online sessions, facilitators covering each topic over two sessions, further developing the role-playing by incorporating patients with COPD, delivering the education sessions over a whole day or block structure, and closely aligning education sessions with the roll out of BE WELL programs at each ACCHS.

### Interviews

Interviews ranged from 15 to 29 min, with six interviews over 20 min. A total of 235 min with AHWs and EPs were recorded. Four themes were identified: 1. Revealing the Aboriginal lung health landscape; 2. Participating in online learning; 3. Structuring the online education sessions; 4. Co-designing with the facilitators. The themes were similar for AHWs and EPs. Variations mainly related to their differing roles within the broader BE WELL project and that none of the EPs were Aboriginal.

### Revealing the Aboriginal lung health landscape

#### Professional experience

Most of the AHWs reported they had only commenced their role as an AHW in the last year, so had limited to no prior experience providing clinical care or delivering health programs. Four AHWs had completed nationally recognised training in Fitness, Mental Health or Aboriginal Health. All AHWs reported no previous education about COPD or experience providing PR programs before participating in the BE WELL project. Several AHWs stated they had not previously heard about COPD.

*“I’ve never had no idea, or understanding of COPD, or what it was about; but you know being a part of this BE WELL Program, I’ve learnt so much.” (AHW_11)*

In contrast with AHWs, EPs had a much longer work experience, but each stated their current employer was the first ACCHS in which they had worked. All EPs reported little to no prior experience providing clinical care or programs for Aboriginal people with COPD.

#### Perspectives of lung health literacy

AHWs and EPs consistently perceived there was low lung health literacy within their respective Aboriginal communities. Some AHWs mentioned when lung disease was discussed, the community focused on asthma or smoking. An AHW stated that when attending smoking cessation appointments with Aboriginal patients, many reported a smoking history of 20 + years, but displayed limited lung health knowledge.

EPs perceived asthma, smoking and asbestosis were discussed in the community, but thought key information about these conditions may be missing. An EP stated lung health is openly discussed due to asbestos mining in the area, with many Aboriginal people dying from asbestos-related lung disease. EPs also spoke about competing priorities for Aboriginal people's attention such as a focus on other chronic health conditions such as diabetes and heart disease rather than chronic lung diseases. This has resulted in Aboriginal patients often presenting to the ACCHS with breathlessness, unsure of the reason for their symptoms.

*"They will come into me and say, Look, I can't breathe very well and I don't know what's going on. And you look at their notes and they're smokers or have a diagnosis of COPD". (EP_01)*

#### Perspectives of access to lung health services

AHWs and EPs perceived Aboriginal people have low access and limited knowledge of lung health services provided by ACCHS and mainstream health services, such as PR. AHWs perceived an ACCHS would be the first point of contact for Aboriginal people with COPD, but acknowledged their ACCHS had low numbers of existing patients diagnosed with COPD. The ACCHS in the BE WELL project had only recently commenced offering PR and most previous clinical discussions related to lung disease focussed on quitting smoking and nicotine replacement therapy.

*“We actually haven't got many clients with [ACCHS] that I know that are diagnosed, but I think they should be diagnosed since starting the BE WELL project.” (AHW_05)*

Since participating in the BE WELL project several AHWs reported they were now able to identify existing ACCHS patients at risk of COPD and able to refer them to PR.

*“And you know, ever since being able to learn more about the lungs and COPD, I'm able to identify the patients when they do come into the clinic.” (AHW_11)*

EPs mentioned delivering PR by their ACCHS was important, considering mainstream health services struggle to engage Aboriginal people because those services are perceived as culturally unsafe.

*“I don't think they go too often [to hospital]. It's not their meeting place.” (EP_01)*

An EP also spoke about needing to work with Aboriginal patients to overcome fear and avoidance of breathlessness during exercise training and maintaining their ongoing participation in an 8-week PR program.

*“The hardest thing for us now [at ACCHS] is to get participants and then get them to regularly attend.” (EP_01)*

EPs also spoke of the need to strengthen the cultural component of lung care by delivering programs on Country away from the clinical environment, spending additional time listening to and allowing Aboriginal people to share their ‘story’, and increasing access to care by improving referral processes within their ACCHS for assessment and management.

### Participating in online learning

#### Experiences of VoIP technologies

Overall AHWs reported the education sessions were successfully adjusted to an online delivery mode using VoIP technologies. AHWs positively described their experience using VoIP technologies as it enabled the educational content to be packaged into small manageable topics around other work demands. This approach supported thorough explanation of each of the seven COPD topics and enabled AHWs to ask questions and revisit educational material.

*"I think the shorter sessions are probably better." (EP_07)*

AHWs mentioned some technical challenges such as limited Wi-Fi connectivity and using a work computer with a broken camera.

*“I enjoyed it. – [Facilitator] went through the sessions really well. [Facilitator] explained every bit, you know, every detail, bit by bit, in the way that I was able to understand.” (AHW_ 11)*

The EPs perception of the use of VoIP technologies varied. Two EPs reported that although online delivery ran smoothly and they got a lot out of the online sessions, their preference would be for face-to-face delivery to enable practical and interactive education. One of these EPs also mentioned they had initially suggested that the education sessions could be provided as a one-day refresher. Positive perspectives about using VoIP technologies were that they were nearly as good as attending in person because information and pictures could be brought up on the computer screen, and questions could always be asked of the facilitators. An EP mentioned using VoIP technologies was better than providing the information quickly face-to-face over two days. From their perspective, the best aspect was having a one week break in between each session to develop yarning scripts. The same EP also reflected on the experience of VoIP technologies for AHWs.

*“I think particularly for the Aboriginal Health Worker they are a little bit quiet and… felt a bit of shame. I suppose both, you know, when you're talking on video conferencing and particularly in the beginning. In the end, they just opened up completely…I think it was really quite good.” (EP_01)*

#### Perspectives of the education sessions and developing yarning scripts

All AHWs reported that engaging with the education sessions, preparing their own yarning scripts, applying the stimulus questions and demonstrating how they would deliver their own yarning sessions to colleagues and the facilitators reassured to them they had interpreted the seven COPD topics correctly. The sessions were reported as being a culturally safe and a self-paced learning environment, where AHWs were driving their own knowledge development, enabling them to confidently design their own yarnings. Several AHWs also spoke about the structured, clear and culturally safe way the scripts they developed enabled them to yarn about complex lung health information simply with Aboriginal people.

*“This isn't like a GP just reading off a piece of paper. Look, we made it into a good discussion, but we [AHWs] made it. So there's feedback, there's input to start more yarnings and kind of branch off each other. Yeah, I think yarning will be a good thing.” (AHW_03)*

For the EPs, the education sessions were reported as helpful and more as a refresher, complimenting their existing lung health clinical skills and strengthening their Aboriginal cultural knowledge. Consistently EPs expressed professional respect for the AHWs, emphasising the value of collegiality, shared decision-making and autonomy. An EP observed that an AHW found it confronting initially to allocate time and to prepare yarning scripts. As a result, the EP supported the AHW to better prepare and deliver their yarning scripts. EPs also reported that the education and yarning sessions enabled them to have confidence that AHWs were seeing good examples of patients with lung disease and were aware of what to look out for clinically.

*“We worked together and I let her present it [the yarning script], cause it's her mob [Aboriginal community], her program. So I was really supporting her delivering the program.” (EP_01)*

### Structuring the online education sessions

#### Perspectives of the education topics

Delivering information into manageable topics was reported to increase comprehension and reduce complexity. Subsequently this scaffolding approach was reported as enabling the AHW to make greater sense of the COPD information.

*“Yeah, especially the medications. That was so far out of my scope at the time.” (AHW_03)*

EPs consistently reported the topics were pitched appropriately and included the right amount and type of information for AHWs, Aboriginal patients and covered everything they needed to know.

*“I think, medication wise, that's one for me that I still definitely need a little bit more assistance with, so that was good to be able to go over them again”. (EP_07)*

#### Perspectives of the resources

AHWs provided consistent positive feedback about the range of resources used during the online sessions. Resources were described as *‘clear’* and *‘culturally safe’*. Resources defined as useful were the pictures, diagrams, booklets, medication charts, PowerPoint slides and videos of the correct use of inhalers and of patients with lung disease. An AHW mentioned practical teaching aides, such as placebo inhalers and spacers were resources that should be used more often in future sessions. Some AHWs stated using videos particularly helped them learn about the symptoms of lung disease.

*“I have not seen anyone with COPD before. Well, I may have, but I didn't know what it is. So, I thought [using videos of patients with lung disease] was great.” (AHW_03)*

All EPs mentioned the resources were useful in their role as supervisors of AHWs. The EPs also perceived the resources as culturally appropriate and helpful. An EP reported the resources had been valuable to their role and ACCHS beyond the BE WELL project and patients with COPD, as the format can be applied across other health programs. Future sessions could be enhanced by incorporating additional resources such as real-life scenarios and videos of patients at the beginning of each session.

#### Perspectives about the frequency and length of the education sessions

AHWs and EPs agreed that the number, length and frequency of each topic and the education sessions were broadly right. Short 30-min sessions were preferred as these allowed staff to maintain focus. An EP mentioned the timing of the education sessions made it hard for them to attend and support AHWs, and that over time attending became more of a chore.

### Co-designing with the facilitators

#### Perspectives of the facilitators’ attributes

AHWs perspectives of the facilitators were overwhelmingly positive. AHWs consistently described the facilitators as ‘*knowledgeable’* and perceived them as *‘culturally competent’* and *‘professionally respectful*’ and *‘understood’* the demands of the AHWs roles and their learning needs. All AHWs felt that their cultural knowledge and perspectives were respected by the facilitators. They also reported the facilitators had created a culturally safe learning environment for Aboriginal organisations and staff to engage in learning by incorporating Aboriginal protocols, such as an Acknowledgement of Country and using resources depicting Aboriginal people.

AHWs described the facilitator (JA) as explaining information in ways the AHWs could understand, which increased AHWs self-reported knowledge. The facilitator had engaged the AHWs in relaxed discussions and provided feedback about the yarning scripts that the AHWs had created. This allowed AHWs to share their perspective, knowledge and yarning approaches openly and deeply. An AHW reported one occasion when the facilitators seemed *‘unapproachable’*. The AHW stated this occurred when minimal staff from their ACCHS attended the first online session which made the session confronting for a lone AHW. The experience resulted in the ACCHS regrouping and identifying how all staff could attend future sessions around clinical responsibilities.

EPs described the facilitators’ attributes similarly to the AHWs. They felt the facilitators supported their learning and encouraged them to contribute to discussions exploring the applicability and adaptation of the educational content and material and the overall BE WELL program to fit the local Aboriginal community context. The EPs also stated the facilitators were responsive to the EPs and AHWs ideas and ways of being, such as how they and the ACCHS worked with community.

*“You know, they were really good at facilitating us to create our own yarn, and also our own ideas for how the program should be run.” (EP_01)*

#### Perspectives of the facilitators’ expectations

Most AHWs described the facilitators’ expectations to attend the online sessions, prepare and present their yarnings as realistic and reasonable. AHWs felt encouraged to make choices and changes to adapt the education sessions.

*“[The facilitators] pretty much gave us free rein…. Well, not to talk us up, but, you know, they [the facilitators] loved what we came up with. So, yeah, like they literally gave us a lot of wriggle room.” (AHW_ 03)*

Two AHWs from different ACCHS highlighted the intensity and demands of the AHW role, while participating in the online education sessions.

*“Because our transport driver called in sick that day, so I had to fill in [and missed a session].” (AHW_08*)

Three of the four EPs mentioned the facilitators were flexible, accommodating and did not expect too much when the EPs were attending the session or helping the AHWs prepare their yarning scripts. The other EP acknowledged the role of an AHW is *‘tough’* and agreed with an AHW at their service that expectations were too high.

*"The expectation felt too much on a couple of the health workers, but that's not the facilitator’s fault. That's just, once again time management, and just being super busy, and juggling lots of things. It can just feel like another thing to have to do." (EP_06)*

#### Perspectives of co-design

All AHWs and EPs agreed co-design occurred. AHWs reported feeling supported by the facilitators and were able to transform the COPD educational material into their own words to better engage the local Aboriginal community.

*“It [using co-design] was just trying to get it more culturally appropriate. So it's not just going to be like the typical GPs talk. Because that’s when you lose people, that's when your gonna lose community. So it's just a yarn.” (AHW_03)*

AHWs simplified the clinical information and incorporated local knowledge, terms and phrases, as well as their personal experiences of the local Aboriginal community. This approach made the yarning resources their own and was reported as relieving the pressure and expectation they initially felt co-designing the educational material. However, they felt that there were more Aboriginal cultural references that could be included in the future as they developed their knowledge and confidence and began to deliver yarnings sessions within their respective BE WELL programs.

*“We haven't as of yet [included local Aboriginal stories or language], because there was a lot of new information for someone learning about COPD.” (AHW_05)*

In addition, EPs commented the facilitators asked enquiring questions of the team about the format, session frequency, timing, topics and whether the local BE WELL teams’ needs were being met, which supported co-design and local adaptation.

## Discussion

The study aimed to explore the experiences of AHWs and EPs from four ACCHS engaged in online education sessions to build knowledge and skills to co-design education ‘yarning’ material for Aboriginal people with COPD. The quantitative survey highlighted the benefits of the online education sessions to build AHWs and EPs capability to co-design COPD yarning resources. The online sessions were also reported as being helpful in allowing AHWs and EPs to support Aboriginal patients with COPD. Overall, the online learning space that was created in the study allowed AHWs to feel their cultural perspectives and professional opinions were valued and respected. Many findings of the survey were crystalised in the qualitative interviews, supporting triangulation [39]. Participants, particularly AHWs, engaged easily with online education using VoIP and reported that the sessions supported self-paced learning, where they could apply their lived experiences and knowledge of their local Aboriginal community to develop reflective, effective and culturally-centred COPD yarning resources. This study adds to the limited PR literature about building health professionals knowledge and skills around the management of people with COPD [15, 40, 41], as well as limited studies evaluating participants’ experiences of participating in co-design [42]. To our knowledge, this is the first study exploring online education for AHWs and EPs to co-design COPD educational ‘yarning’ material for Aboriginal people with COPD guided by 8 Ways of learning.

### Online education

Advancements in technology-enabled learning are revolutionising face-to-face education towards online platforms and classrooms, which has potential to support learning and self-management of patients with COPD, PR programs broadly, as well as how health professionals access and engage with education [41, 43]. The online education sessions were designed to be participant-centred, responding to AHWs’ and EPs’ training needs and placed value on the use of co-design and 8 Ways of learning. The value of incorporating an Aboriginal pedagogy framework to improve culturally safe and accessible mainstream services is being realised within the NSW public health sector, with the first study published focusing on introducing 8 Ways of learning guided by participatory action research to shape project and program design [20]. Variations were made to the format and structure of the online education sessions to align with AHWs’ and EPs’ socio-cultural, professional and the contexts of each of the four ACCHS. Of note was how positively AHWs perceived online education. The AHWs engaged with the learning material and teaching approaches, which enabled a supportive and flexible learning environment to be created. It has been reported in other studies that Aboriginal people are early adopters of technology and partners in digital health programs and research [44, 45]. In this study it was reported that using VoIP technologies enabled information to be paced and different teaching techniques applied to thoroughly explore specific aspects of COPD. The structure of the online education sessions allowed adequate time from when AHWs and EPs received new COPD knowledge, adapted this information to the local Aboriginal community and presented their own yarning sessions. The structured scaffolding approach of building knowledge across the seven topics enabled complicated information to be easily understood by the AHWs, even in topics, such as medications that they initially believed were outside their scope and ability [28].

### Culturally safe learning environment

AHWs and EPs highlighted that the online education sessions were culturally safe spaces to ask questions and seek advice and feedback. AHWs expressed they experienced professional independence and autonomy and collaborated with facilitators and EPs, which supported their knowledge development and facilitated local accountability to confidently co-design their own yarning material. EPs specifically found the online education sessions which used 8 Ways of learning culturally helpful, especially for their supervisory role with AHWs. The sessions also further enhanced their existing clinical knowledge and experience in managing people with COPD.

The EPs’ cultural competence and support of AHWs was evident. This was important as AHWs within the ACCHS sector have reported negative experiences working with allied health professionals [46]. EPs consistently expressed cultural respect and professional humility towards the value of AHWs role within the ACCHS and their communities. EPs actively encouraged AHWs to apply their professional and personal autonomy, knowledge and skills. EPs listened to AHWs’ perspectives when they explored COPD from an Aboriginal worldview, and openly shared their own clinical interactions working with and understanding Aboriginal patients lived experience of COPD, and reasons they may choose to engage (or not engage) in services provided by ACCHS. As a result, AHWs, supported by EPs, successfully co-designed their own COPD yarning resources which were reflective of the Aboriginal communities they serve. AHWs also self-reported improved knowledge, skills and ability in providing care for Aboriginal people with chronic lung disease. These findings aligned with Browne et al., that highlighted the importance and benefit of an equal partnership and working with culturally competent allied health professionals who display reciprocity of two-way learning and who are committed to building AHWs’ capabilities [46]. These findings also acknowledge the importance of using 8 Ways of learning and investing in similar frameworks that equally value and incorporate Western and Indigenous knowledges and systems in health research and education, such as *Both-Ways Seeing or Two-Eyed Seeing* [47–50]. While there was an integration of Western and Indigenous knowledges and systems, the topics covered during the online education sessions maintained a Western understanding of health and disease.

### Aboriginal landscape

Overall, the study revealed COPD in Aboriginal communities is poorly understood and could be under-detected and that services for managing COPD, such as PR, are underdeveloped. AHWs and EPs perceived limited COPD health literacy and low access to lung services within ACCHS or mainstream health services by the local Aboriginal community. Although limited studies and no national data are available, it has been reported that Aboriginal people are highly likely to be at risk of poorer health literacy [51, 52], and low uptake of PR [9]. AHWs and EPs consistently perceived mainstream services were unable to deliver culturally safe lung health services for Aboriginal people, which is reflected in other studies about access to mainstream health care [11]. Low knowledge and awareness of COPD and PR has been found in the mainstream rural and remote health workforce [15], and was mirrored with AHWs and EPs in this study. Through the online education sessions AHW’s increased their self-reported knowledge, awareness and management of COPD, and their skills to support the early detection and diagnosis of COPD in Aboriginal people. ACCHS have a long and successful history of delivering high quality culturally safe care, with AHWs playing a critical role as the first point of contact and cultural brokers, facilitating Aboriginal peoples’ access to health promotion, education, and clinical services [11, 17]. Whilst there is an imperative to improve the cultural safety of mainstream PR programs, an opportunity exists for the ACCHS sector to lead COPD service provision, workforce development and implement PR that better meets the needs of Aboriginal people in the community and increases access to care [53].

### Local adaptation

Consistently, cultural adaptation occurred around ways of talking and communicating (‘yarning’) to make COPD information and engagement with the material conversational. Developing research skills of AHWs is particularly important during Aboriginal research [54]. AHWs, even those new to the profession, bring significant knowledge and experience of their community and how the community accesses health care and engages with health education and programs [55]. Our research confirms that in many instances, AHWs drew from their lived experiences and engagement with their local Aboriginal community to adapt complex clinical information to co-design COPD yarning resources. AHWs incorporated their own Aboriginal worldviews, phrasing and experiences to fit the context of the ACCHS and reflect the local Aboriginal community, which allowed AHWs to translate, not replicate the material verbatim. Further, the yarning scripts developed aligned with how the AHWs and the ACCHS engage and communicate health information with the local Aboriginal community. The value of cultural adaptation or Indigenising health care, teaching and education is broadly known [20, 21, 56]. It is anticipated that as AHWs work more closely with patients living with COPD who attend the BE WELL PR program, and they begin delivering yarning sessions, inclusion of Aboriginal language, stories and deeper cultural understanding related to COPD may occur.

### Challenges

The study highlighted some challenges for implementing online education sessions. Some AHWs and EPs reported pressures participating in the study and attending the online sessions while meeting other work responsibilities, staying engaged over time, and preparing the yarning scripts too far in advance of the ACCHS commencing their BE WELL PR Program. These findings are consistent with Farnbach et al. [55], which revealed the increased pressure experienced by staff involved in the dual roles of clinical practice within the community and engaging with research [55]. This pressure of dual roles was consistently reported by one ACCHS, likely illustrating when co-design principles were poorly applied, and the timing, structure and format of the online education sessions did not entirely align with their needs. This feedback highlights the challenge for participants working within an ACCHS to find time to engage in training while maintaining their clinical roles and community responsibilities. Co-design emphasises the importance of sharing power and maintaining equal partnerships with participants, which can be a delicate balance, considering time pressures of participants [42]. This feedback emphasises the importance of using participatory engagement and shared decision making to mitigate pressures for participants to engage in training, which are more effective than top-down approaches [56].

### Strengths and limitations

Initial coding was conducted independently by three researchers (DM, JA, SD). The results represent the experiences of AHWs and EPs by using their voices as study participants. The engagement of Aboriginal and non-Aboriginal members of the research team during the design, delivery and analysis strengthens the inclusion of Aboriginal perspectives and meaning of the data. Two members of the research team (JA and DM) facilitated the online education sessions with close and ongoing involvement with participants. They developed deep knowledge about the insights, benefits and strengths of the study, and identified ‘objective distance’ was required during data collection, because both were close to the study and mindful of the potential positive and negative effects on research quality. To reduce researcher bias, an independent member of the research team (KG) with no contact with the participants during the online education sessions conducted the qualitative interviews using VoIP technologies. It is acknowledged additional research is required to validate the impact of VoIP technologies on the quality of participants’ experiences of qualitative data collection [35]. Participants completed the survey and interview between two to eight weeks following the online education sessions, the latter might have led to recall bias. Although staff from four diverse NSW-based ACCHS participated in the online education sessions and contributed data, findings may not be generalisable to other ACCHS. However, the data provide insights that online education can be successful in building ACCHS workforce capacity and enabling co-design of culturally safe education resources. As such the online environment may provide greater potential for scale-up of the education component of PR across the ACCHS sector.

Future research should explore whether online education of AHWs and EPs to develop yarnings about COPD management, improves patient health outcomes, enables behavioural changes and increases access to culturally safe PR. Future researchers should also be conscious of the importance of an iterative and flexible process, with a genuine commitment to establishing and maintaining equal partnerships with participants.

## Conclusion

Online education using co-design and 8 Ways of learning was rated highly by AHWs and EPs for improving COPD knowledge and valuing cultural perspectives. The use of co-design principles and 8 Ways of learning with AHWs and EPs supported the cultural adaptation of COPD resources for Aboriginal people with COPD. The findings demonstrated that building culturally safe learning environments can provide effective online learning spaces that increase knowledge, skills and confidence and promote effective participation and collaboration. Key to these findings was respect for AHWs’ cultural knowledge and lived experiences and deeply valuing Aboriginal ways of learning and communicating through yarning.

## Supplementary Information

**Additional file 1.** Survey. BE WELL online education participant survey tool.

**Additional file 2.** Interview questions. BE WELL online education participant interview questions.

## Data Availability

The data that support the findings of this study are available on request from the corresponding author [DM]. The data are not publicly available due to them containing information that could compromise research participant privacy and consent.

## Abbreviations

COPD: Chronic obstructive pulmonary disease
AHWs: Aboriginal health workers
EPs: Exercise physiologists
ACCHS: Aboriginal Community Controlled Health Services
PR: Pulmonary rehabilitation
BE WELL: Breathe Easy, Walk Easy, Lungs for Life
ANZCTR: Australian New Zealand Clinical Trials Registry
VoIP: Voice over Internet Protocol—mediated technologies
