# Supplementary material for: A mixed methods study of Aboriginal health workers’ and exercise physiologists’ experiences of co-designing chronic lung disease ‘yarning’ education resources
Source: BMC Public Health. 2023 Mar 31;23:612. doi: 10.1186/s12889-023-15508-y (PMC10063331; doi:10.1186/s12889-023-15508-y)
Supplement: Supplementary file 1 — Additional file 1. Survey. BE WELL online education participant survey tool. [file 12889_2023_15508_MOESM1_ESM.pdf]

Additional File 1_Supplementary_ Survey

Role:
Time in current position:
Organisation:

Aboriginal	Yes ☐	No ☐
Torres Strait Islander	Yes ☐	No ☐
Both Aboriginal and Torres Strait Islander	Yes ☐	No ☐

**1**	The yarning sessions were easy for me to understand	Always……………………………….	☐
		Mostly……………………………….	☐
		Sometimes………………………….	☐
		Rarely……………………………….	☐
		Never……………………………….	☐

If you would like to, please give a reason for your choice to question 1:

____

**2**	What did you think about the use of online zoom for the yarning education?	Very good…………………………..	☐
		Good………………………………….	☐
		Average…………………………….	☐
		Poor………………………………….	☐
		Very poor………………………….	☐

If you would like to, please give a reason for your choice to question 2:

____

**3**	Were you able to ask questions when you wanted to during the online zoom yarning education?	Always……………………………….	☐
		Mostly……………………………….	☐
		Sometimes………………………….	☐
		Rarely……………………………….	☐
		Never……………………………….	☐

If you would like to, please give a reason for your choice to question 3:

____

**4**	The yarning sessions had enough information about each topic	Strongly agree……………………….	☐
		Agree………………………………….	☐
		Unsure……………………………….	☐
		Disagree…………………………….	☐
		Strongly disagree…………………….	☐

If you would like to, please give a reason for your choice to question 4:

____

**5**	The yarning sessions helped me understand chronic lung disease	Strongly agree……………………….	☐
		Agree………………………………….	☐
		Unsure……………………………….	☐
		Disagree…………………………….	☐
		Strongly disagree…………………….	☐

If you would like to, please give a reason for your choice to question 5:

____

**6**	The yarning sessions helped me to gain knowledge and skills on how to help patients/clients manage their lung disease	Strongly agree……………………..….	☐
		Agree…………………………………	☐
		Unsure……………………….………	☐
		Disagree…………………………….	☐
		Strongly disagree……………………	☐

If you would like to, please give a reason for your choice to question 6:

**7**	The resources provided were helpful for use with patients/clients	Strongly agree……………………..….	☐
		Agree…………………………………	☐
		Unsure……………………….………	☐
		Disagree…………………………….	☐
		Strongly disagree……………………	☐

If you would like to, please give a reason for your choice to question 7:

**8**	The yarning sessions were too long	Strongly agree……………………..….	☐
		Agree…………………………………	☐
		Unsure……………………….………	☐
		Disagree…………………………….	☐
		Strongly disagree……………………	☐

If you would like to, please give a reason for your choice to question 8:

**9**	The yarning sessions were too often	Strongly agree……………………..….	☐
		Agree…………………………………	☐
		Unsure……………………….………	☐
		Disagree…………………………….	☐
		Strongly disagree……………………	☐

If you would like to, please give a reason for your choice in question 9:

**10**	The research team (Jenny and David) who presented the yarning valued my cultural perspectives about the topics	Always……………………………….	☐
		Mostly………………………………..	☐
		Sometimes…………………………..	☐
		Rarely………………………………..	☐
		Never………………………………..	☐

If you would like to, please give a reason for your choice to question 10:

**11**	The yarning sessions that I prepared and delivered helped me to understand the topics better	Always……………………………….	☐
		Mostly………………………………..	☐
		Sometimes…………………………..	☐
		Rarely………………………………..	☐
		Never………………………………..	☐

If you would like to, please give a reason for your choice to question 11:

**12**	I felt encouraged to include my cultural knowledge into the topics that I presented	Always………………………………	◻
		Mostly……………………………….	◻
		Sometimes…………………………..	◻
		Rarely……………………………….	◻
		Never………………………………..	◻

If you would like to, please give a reason for your choice to question 12:

**13**	I felt that my opinions about the changes that could be made to the topics were valued	Always………………………………	◻
		Mostly……………………………….	◻
		Sometimes…………………………..	◻
		Rarely……………………………….	◻
		Never………………………………..	◻

If you would like to, please give a reason for your choice to question 13:

**14**	The feedback I received from the research team on the topics I presented was helpful	Always………………………………	◻
		Mostly……………………………….	◻
		Sometimes…………………………..	◻
		Rarely……………………………….	◻
		Never………………………………..	◻

If you would like to, please give a reason for your choice to question 14:

**15**	Overall, I found the yarning sessions useful	Always………………………………	◻
		Mostly……………………………….	◻
		Sometimes…………………………..	◻
		Rarely……………………………….	◻
		Never………………………………..	◻

If you would like to, please give a reason for your choice to question 15:

**16**	The topics I found most useful were?	Understanding the lungs	_
		What can go wrong with the lungs	_
	**Please rank in order from 1-7**	Inhaled medications and how to take them	_
		Why exercise is important	_
	**(1 = most useful, 7 = least useful)**	How to manage breathlessness	_
		Healthy Eating	_
		Managing Anxiety, Depression & Stress	_

If you would like to, please give a reason for your choice to question 16:

Final comments

1. What did you like most about the Yarning education sessions?
2. What part(s) of the Yarning education sessions do you think needs improving?
3. Do you have any further comments about the education sessions?
