# Supplementary material for: A mixed methods study of Aboriginal health workers’ and exercise physiologists’ experiences of co-designing chronic lung disease ‘yarning’ education resources
Source: BMC Public Health. 2023 Mar 31;23:612. doi: 10.1186/s12889-023-15508-y (PMC10063331; doi:10.1186/s12889-023-15508-y)
Supplement: Supplementary file 2 — Additional file 2. Interview questions. BE WELL online education participant interview questions. [file 12889_2023_15508_MOESM2_ESM.pdf]

Additional file 2_Summlemetary_Interview Questions

• What has been your experience delivering health care services with Aboriginal peoples?
• What do you think is the level of awareness and understanding about chronic lung disease in the local Aboriginal community?
• What was the original agreement between yourself and the facilitators about developing the education sessions?
• What did you think about using an online platform for the education sessions?
• What did you think about the way each week's sessions were delivered?
• How were you motivated to attend the online sessions?
• Were the resources helpful to prepare your own yarning sessions?
• What did you think about the number, frequency and length of the education sessions?
• How did the yarning sessions impact your knowledge about chronic lung disease?
• How did the yarning sessions help you support patients manage chronic lung disease?
• How did the presenter respect your cultural knowledge?
• How did the facilitators engage you in discussions?
• How did the facilitators respect your knowledge and opinions?
• What were the facilitators expectations of you to participate in the education sessions?
• What impact do you think the yarning sessions, scripts and resources you prepared will have on your AMS and the local Aboriginal community?
